# Remarks on the Influence of Climate, Situation, Nature of Country, Population, Nature of Food, and Way of Life; on the Disposition and Temper, Manners and Behaviour, Intellects, Laws and Customs, Form of Government, and Religion of Mankind

**Published:** 1781-06

**Authors:** 


III. *Remarks on the influence of climate, ſituation, nature of country, population, nature of food, and way of life; on the diſpoſition and temper, manners and behaviour, intellects, laws and cuſtoms, form of government, and religion of mankind. By* William Falconer, M. D. F. R. S. 4to. Dilly, *London*, 1781. 552 pages, 1l. 1s. in boards.

VEGETABLE, and even animal productions, ſeem to be limited by nature to certain ſituations. Man, however, appears to be an exception to this rule, and to be enabled to ſubſiſt in every climate. He reigns with the lion and the tyger under the Equator, and aſſociates with the bear and rein-deer beyond the Polar Circle.

Nor is man leſs capable of ſubſiſting on a great variety of aliments, than he is able to endure a great difference of climate; the former of which circumftances, as well as the latter, is very properly adduced by naturalifts, as a great prefumption that he was intended by nature to inhabit every part of the world. But notwithftanding this affiftance afforded by nature, it may be juftly doubted if this univerfality of the human fpecies be not owing more to his rational faculties, which enable him to fupply the defects, and correct the exuberances of particular climates and fituations, than merely to his animal formation.

But although man is enabled to fubfift by means of thefe fuccours from his rational faculties, he is ftill liable to be confiderably affected, both in his body and mind, by external circumftances, fuch as climate, fituation, &c. To enumerate fome of thefe, with their general effects, is the purpofe of the work before us, which the author with diffidence offers to the public, not as a complete difcuffion of the fubject, but only as a collection of fuch obfervations as occurred to him on confidering it.

The volume is divided into fix books, correfponding with the fubjects mentioned in the title. Of thefe, the firft and the laft are by far the moft confiderable; the former, on the effect of of climate, occupying 166 pages, and the latter, on the influence of way of life, 295 pages of the work. Under each head we meet with a great number of interefting remarks, the refult of extenfive reading; but as the work, from the nature of its plan, is calculated for the philofophical rather than the medical reader, we fhall content ourfelves with felecting from this variety of matter the following obfervations on diet.

There is no nation, our author obferves, that live entirely upon either animal or vegetable food; but all ufe in fome meafure a mixture of both. The Brachmans, who are faid to live on a vegetable diet, eat milk, which is partly of an animal nature; and the abftinence they practife appears to be too rigid for even fo hot a climate, as they are in general meager, weak and fickly, labouring under a conftant diarrhœa, and feveral other diforders. On the other hand, the Laplanders are faid to live on animal food only; but this has been contradicted by Linnæus, who fays, that befides milk, which they alfo take four, they ufe fome of the fpecies of arum, of the marfh, trefoil, and other plants very copioufly; fo that there is no inftance of any nation living entirely on either of thefe

 diets diets, though there are ſeveral which vary the proportion of them reſpectively.

Dr. Falconer remarks, after Haller, that animal food is much more nutritious than vegetable; both as containing a greater quantity of nouriſhment, and as this nouriſhment is more eaſily extracted. It likewiſe affords a greater ſtimulus, and of courſe is more perſpirable than vegetable food; but at the ſame time it loads and oppreſſes the body, and requires the conſtant repetition of a fever to throw it off; which tends greatly to wear out the conſtitution.

Theſe properties, we are told, are more remarkable in the fleſh of wild than of tame animals; in the carnivorous than in the herbivorous; in the old than in the young; in ſuch as are eaten raw than in thoſe that are dreſſed with fire; and in ſuch as are killed in the blood, than in ſuch as are bled to death. It is true, that no carnivorous animals are at preſent uſed in diet among Europeans; but Dr. Shaw ſays that the lion was eaten at Algiers, and that it taſted like veal. Hippocrates mentions the fleſh of the dog and of the fox in his treatiſe on diet; and the Romans ate the fleſh of rats, which they fed on vegetable food only, as the

Otaheiteans do the dogs they uſe for the ſame purpoſe.

The effects of raw meat on the diſpoſition of animals is inſtanced in the greater fierceneſs and rapacioufneſs of dogs fed in this manner, than of ſuch as eat their victuals dreſſed. From this proceeds the great ferocity of butchers dogs; and the tendency of this food to render them miſchievous is remarked in thoſe that are apt to kill ſheep, which after having once taſted the raw fleſh, are with great difficulty reclaimed. Beaſts of prey probably owe their ſuperior fierceneſs in a good meaſure to their food, which is always raw, killed in the blood, and moſtly of the wild kind. The ſame, adds our author, holds good of mankind. Thoſe nations who live by hunting are in general of a cruel diſpoſition.

In conſidering the effects of animal diet upon the underſtanding and intellects, he ſpeaks of it as being evidently adverſe to the exertions of genius, ſentiment, and delicate feelings, as well as to deep mental reſearches. At the ſame time he thinks it may be better adapted to the common buſineſs of life, than a diet which produces a greater degree of ſenſibility.

With

With regard to vegetable food, our author obſerves, that its producing a more ſpare habit of body than animal diet, is probably owing, not only to its containing leſs nutriment, but alſo to the acidity which prevails in all vegetable ſubſtances. Some compenſation is, however, made for its being leſs nutritive, by its being capable of being taken in much larger proportion than animal food. In hot countries, the climate renders ſeveral fruits ſafe, and even neceſſary, in ſuch quantities as would be highly dangerous with us. People in Perſia, according to Chardin, will eat at once, or at leaſt in twenty-four hours, thirty-five pounds weight of melon.

It appears, however, that notwithſtanding the greater quantity in which vegetables may be taken, they ſtill require ſome ſtimulus to be joined with them, in order to render them fit for the purpoſes of aliment. This is very bountifully beſtowed by nature in thoſe climates where vegetable food is moſt uſed, and conſiſts of pungent aromatic plants, which though of themſelves nearly deſtitute of nouriſhment, correct the bad qualities, and ſupply the defects of thoſe vegetables that are more nutritious.

Our

Our author defcribes the effects of vegetable food on the difpofition, &c. of mankind, as being exactly the reverfe of thofe produced by animal diet. He mentions, after Arbuthnot, that the fierceft lions have been rendered tractable and docile by being fed upon vegetable food, and obferves, that the fame writer fpeaks of feveral inftances that had fallen under his own obfervation, of irafcibility of temper, in the human fpecies, being fubdued by a vegetable regimen. In proof of its being favourable to mental exertions, he quotes the anecdote related by Cheyne of Sir Ifaac Newton, who was fo fenfible of the unfavourable operation of animal food, that during the time of his writing his treatife on optics, which is generally thought to be the work wherein his genius difplayed itfelf in its fulleft force, he lived on a vegetable diet only, and that extremely fimple and rigid.

The author agrees with Haller in confidering fifh as holding a kind of middle rank between animals and vegetables. They are, he obferves, in general lefs putrefcent, and alfo lefs nutritive than flefh meats, and produce alfo lefs red blood and ftrength of body. He does not undertake to determine, whether the idea fuggefted by fome writers, writers, that a fiſh diet is more prolific than one of either animals or vegetables, is well-founded or not.

He divides liquid food into the fermented and unfermented. Of the latter he takes water as an inſtance. He obſerves, that from its poſſeſſing no ſtimulant quality, the drinking of it has a tendency to render the temper even and regular; and that as it does not diſorder the underſtanding, thoſe who drink it are apt to acquire a habit of ſecrecy and reſerve. He likewiſe conſiders it as favourable to morality.

Speaking of fermented liquors, he remarks, that they produce an increaſed flow of ſpirits, and have alſo the effect of opening the mind. To the greater uſe of theſe liquors he aſcribes the greater frankneſs and hoſpitality of the Northern nations. Another effect aſcribed to fermented liquors, is that of inſpiring genius and ſentiment, eſpecially of the poetical kind. This, at firſt ſight, might ſeem ludicrous; but is ſeriouſly aſſerted by ſeveral grave writers, and is, he believes, in ſome degree founded upon truth.

Malt liquors, we are told, poſſeſs in many reſpects the ſame qualities with wine, but have not the ſame reputation for improving the intellects. This our author afcribes to their greater vifcidity, which prevents the effects of the fpirituous part upon the nervous fyftem; to its being more nutritious; and laftly, to its having but little of the acid which accompanies wine, and is of great efficacy in caufing the latter to pafs off quickly by the fecretions.

Diftilled fpirits might appear to have nearly the fame effects with wine, as being very thin, light, and poffeffing nearly the fame powers of the fpirituous kind; but in reality wine and fpirituous liquors differ very much from one another. Diftilled fpirits, the author obferves, want the acid of wine, and therefore remain longer in the body and are more inflammatory. They are alfo more narcotic, and debilitate the nervous fyftem more than wine. They are likewife deftitute of fixed air, to which wine in a great meafure owes its invigorating and chearing qualities.

Dr. Falconer concludes this part of his work with fome remarks on tea, the ufe of which he thinks may perhaps be lefs prejudicial in the hot climates of China and India, than it is in ours. But the noxious qualities of this plant, he adds, are not unknown, even in its native countries. The Japanefe are fubject to the diabetes, and to consumptive disorders resembling the atrophy, from its use; and the Chinese, it is said, are so sensible of these consequences, that they rarely drink green tea at all, which is the most remarkable for these effects.

