# Errata

**Published:** 1781-06

**Authors:** 


					-p. 40%
1. J2, for llv is. read las.
END of VOL. I.

				

